# Bilateral paralysis of peroneal nerve after COVID-19 disease: a case report

**DOI:** 10.1186/s12883-022-02613-5

**Published:** 2022-03-14

**Authors:** Natalia Morawiec, Daria Chyra, Adrianna Boroń, Bożena Adamczyk, Jerzy Jaroszewicz, Barbara Sobala-Szczygieł, Monika Adamczyk-Sowa

**Affiliations:** 1 Department of Neurology, Faculty of Medical Sciences in Zabrze, Medical University of Silesia in Katowice, ul. 3 Maja 13-15, 41-800 Zabrze, Poland; 2 Department of Infectious Diseases and Hepatology, Medical University of Silesia in Katowice, ul. Aleja Legionów 49, 41-902 Bytom, Poland

**Keywords:** COVID-19, SARS-CoV 2, Paralysis, Peroneal nerve, Neurological complications

## Abstract

**Background:**

COVID-19, caused by a novel coronavirus SARS-CoV 2 has rapidly developed into pandemic. This infectious disease affecting mainly respiratory system may cause multiple systemic disorders. With increasing number of new infected patients there are more and more cases with neurological complications secondary to COVID-19.

**Case presentation:**

Here we present a case of 67-years old Polish male with previously no comorbidities, who has developed bilateral paralysis of peroneal nerve after SARS-CoV 2 infection. Prior to the hospitalization he presented cough and fever and weakness. RT-PCR was reported positive for COVID-19 infection. Then he developed pneumonia and respiratory failure with bilateral lung consolidations on radiological examination. Laboratory findings revealed elevated levels of D-dimer, CRP, AspAT, GGTP, PCT and serum glucose. After discharge from hospital he was diagnosed with thrombophlebitis and prediabetes on follow-up visits. Due to problems with walking, numbness of toes and involuntary muscle spasms in hands, the patient went to the Neurological Outpatient Clinic. After neurological examination bilateral paralysis of peroneal nerve was revealed.

**Conclusions:**

In this report we want to highlight one of the unexpected presentations of SARS-CoV 2 infection and emphasize the importance of neurological examination in COVID-19 patients.

## Background

Coronavirus disease 2019 (COVID-19), caused by Severe Acute Respiratory Syndrome Coronavirus 2 (SARS-CoV 2), has undoubtedly become the most serious health threat of the 2020 year. In the beginning, it was known as severe respiratory infection with its typical symptoms such as fever, cough, fatigue, dyspnea and complications connected with pneumonia and acute respiratory distress syndrome [1]. With the increasing number of confirmed cases more and more possible manifestations of new coronavirus infection are described, such as cardiovascular or neurological complications [2, 3]. In this report, we describe a case of 67-years-old male who developed bilateral paralysis of peroneal nerve and thrombophlebitis after COVID-19. This manifestation of the disease seems to be unexpected and unusual. We want to highlight the importance of neurological complications secondary to COVID-19.

## Case presentation

**Fig. 1:**
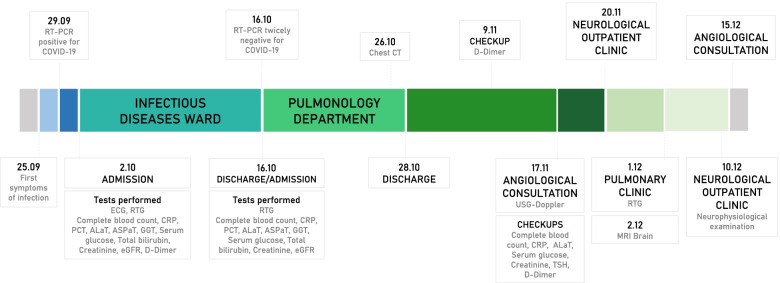
Timeline of the events

**Fig. 2:**
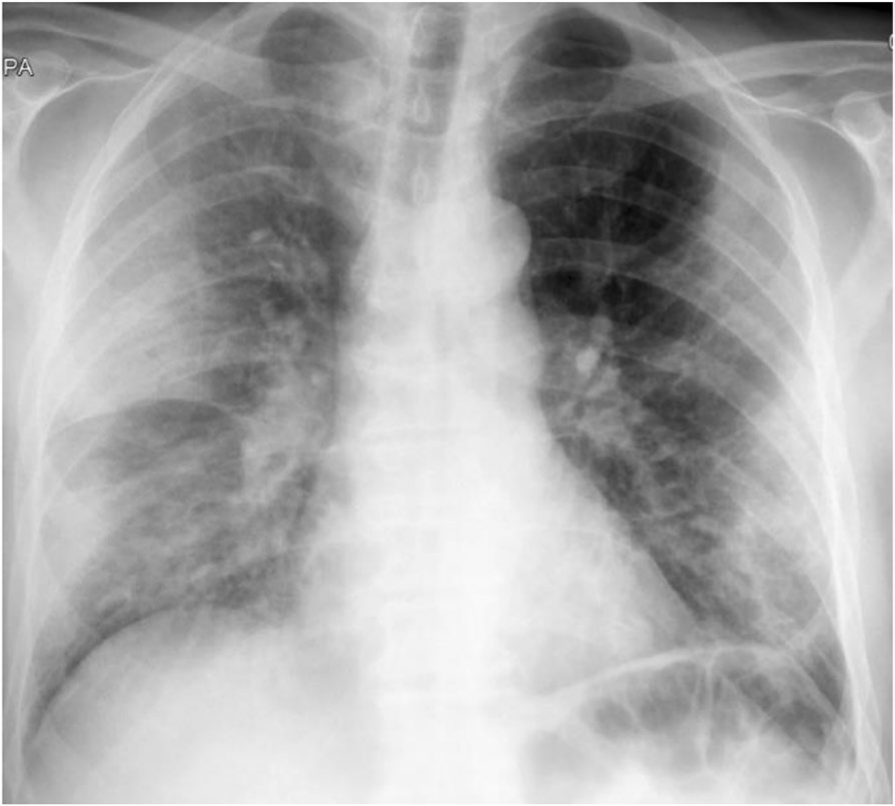
Chest X-ray on admission to the Infectious Diseases Ward which revealed the suspicion of bilateral pneumonia and the symptom of a frosted glass

**Fig. 3:**
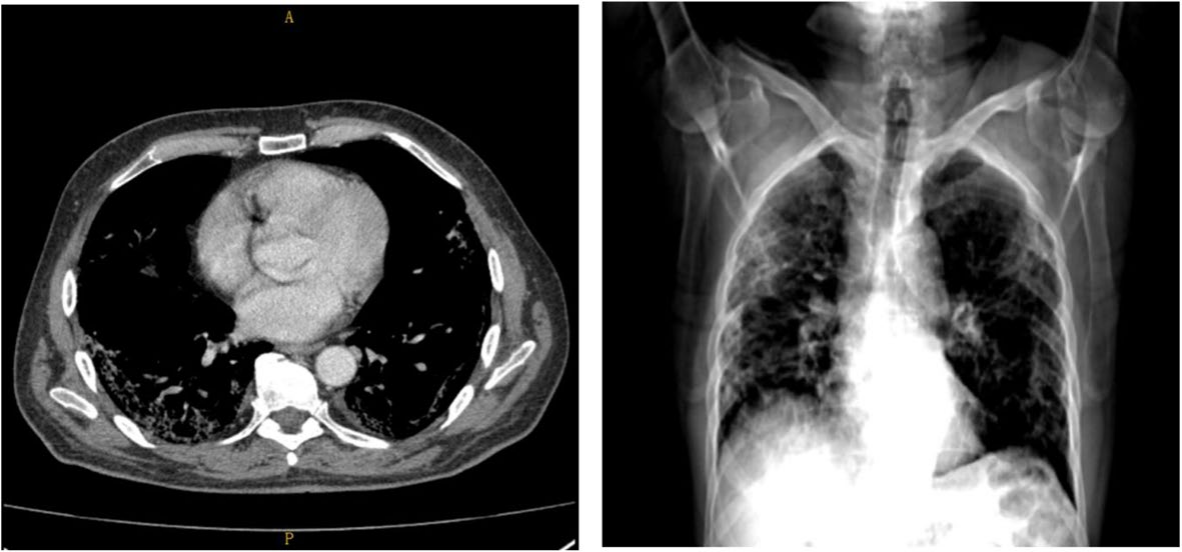
Spiral computed tomography of the chest shows irregular and advanced interstitial changes and ground-glass opacities in peripheral areas of lungs

A 67-years-old Polish male patient was admitted to the Infectious Diseases Ward in Chorzów, Poland with symptoms of COVID-19 pneumonia (Fig. 1). He had no comorbidities or any relevant information in his medical or family history. One week prior to hospitalization, the patient suffered from cough, fever. He felt weak and had problems with everyday activities. Before the admission he was tested positively for SARS-CoV 2. On admission physical examination revealed fever, tachycardia and oxygen saturation of 89% while breathing ambient air. Lung auscultation revealed crackles at the right lung. Chest X-ray showed the suspicion of bilateral pneumonia (Fig. 2). The symptom of a frosted glass on CT of the chest has also confirmed this diagnosis (Fig. 3). Laboratory data on admission demonstrated elevated levels of: D-dimer (2,97 µg/ml, the reference range: 0–0.5 µg/ml), C – reactive protein (CRP) – 186,80 mg/l (the reference range: 0–5 mg/L), aspartate transaminase (AspAT) – 68 U/L (the reference range: 10–50 U/L) and gamma-glutamyl transferase (GGTP) – 84 U/L (the reference range: 10–71 U/L), the procalcitonin (PCT) level of 0,34 ng/ml and random serum glucose – 196 mg/dl (the reference range: 70–99 mg/dl) (Table 1). Electrocardiogram (ECG) showed onset of biphasic T-wave in V6 and left axis deviation. ECG changes were not clinically significant. The patient was treated with remdesivir (200 mg on Day 1 and 100 mg daily for 9 days), dexamethasone (6 mg per day), empiric antibiotic therapy (ceftriaxone – 2 g per day), low molecular weight heparin (LMWH) (40 mg/0,4 ml per day), passive oxygen therapy with a face mask. The symptomatic treatment was also applied (ketoprofen 50 mg twice a day).

**Table 1:** Laboratory data on admission to Infectious Disease Department and Pulmonology Department

Laboratory Findings (The reference range)	Infectious Diseases Department		Pulmonology Department	
C – reactive protein (0–5 mg/L)	**186,8**		**11,15**	
Procalcitonin (< 0,5 ng/mL – low risk of sepsis or septic shock > 2 ng/mL – high risk of sepsis or septic shock)	0,34		-	
Aspartate Aminotransferase (10–50 U/L)	**68**		20	
Alanine aminotransferase (10–50 U/L)	37	23	47,3	
Gamma–Glutamyl Transferase (10–71 U/L)	**84**	-	-	
Serum glucose (70–99 mg/dL)	**196**	**217**	**188,3**	
Total bilirubin (≤ 1,2 mg/dL)	0,46	0,61		
Creatinine (0,67–1,17 mg/dL)	0,97	0,78	0,84	
eGFR (> 60 ml/min/1,73 m^2^)	82,18	106,16	> 60	
D – Dimer (0–0,5 µg/mL)	**2,97**	**10,815**	**3,89**	
Red blood cell count (4,63–6,08 × 10^6^/uL)	4,80	**4,26**	**4,59**	5,11
Haemoglobin (13,7–17,5 g/dL)	14,1	**12,4**	**13,5**	14,9
Haematocrit (40,1–51,0%)	41,3	**36,4**	**40,0**	43,6
White blood cell count (4,23–9,07 × 10^3^/uL)	7,78	**15,36**	**13,10**	**13,64**
Lymphocytes (1,32–3,57 × 10^3^/uL)	0,87	2,00	3,34	**4,33**
Neutrophils (1,78–5,38 × 10^3^/uL)	**6,48**	**10,96**	**8,34**	**8,17**
Platelets count (163–337 × 10^3^/uL)	224	**347**	**151**	**146**

### Hospitalization at the pulmonology department

Testing for SARS-CoV-2 by the method of reverse transcriptase-polymerase chain reaction in real time (RT-PCR) was negative twice. Considering that, on the 15th day of hospitalization, the patient was transferred to the Pulmonology Department in Chorzów for further treatment of pneumonia with respiratory failure (Fig. 1). The patient suffered from shortness of breath. Auscultation revealed crackling noises at the bases of the lungs. Gasometry showed hypoxemia on admission. CXR revealed bilateral lung consolidations. Laboratory data on admission demonstrated: CRP – 11,15 mg/l, AspAT – 20 U/L and random serum glucose – 217 mg/dl. A spiral computed tomography (CT) of the chest was performed. It showed irregular and advanced interstitial changes and ground-glass opacities in bilateral and peripheral areas of lungs (CO-RADS 4) (Fig. 3). The patient received systemic steroids (dexamethasone – 20–10-0 mg) clarithromycin (500 mg every 12 h), bronchodilators, LMWH (40 mg/0,4 ml per day) and passive oxygen therapy.

The patient’s respiratory symptoms and general condition improved. He claimed that he feels much better. He had no dyspnea. There were no significant abnormalities in the condition of his skin or bedsores. The patient did not developed any hospital-aquired infections. He only lost his weight—5 kg from the state before the disease. There was no muscle wasting or any signs of peripheral neuropathy. Lung auscultation 26 days after hospital stay revealed no rhonchi and the patient was discharged from the hospital with recommendation to continue steroid therapy (prednisone 20–10-0 mg with scheme of doses reduction: 2–12.12.2020 10–10-0 mg, 13–23.12.2020 10–5-0 mg, 24–30.12.2020 5–0-0 mg).

### Follow-up at the pulmonology clinic and thromboembolic complications

Due to the patient complaints of both lower limbs oedema which endure for a few weeks and elevated level of D-dimer (3,89 µg/ml, the reference range: 0–0.5 µg/ml) on 20th day after discharge from hospital Doppler ultrasonography (USG) of the lower extremities was performed and revealed right-sided thrombosis of the popliteal vein, the distal part of superficial femoral vein and proximal part of posterior and anterior tibial vein. He was diagnosed with thrombophlebitis and impaired glucose tolerance (according to OGTT). The control laboratory tests after discharge from the Pulmonology Department are presented in Table 1.

The follow-up Doppler USG of lower extremities was performed and showed progressive recanalization of previously thrombosed veins. Rivaroxaban (20 mg per day) was administered on 15.12.2020 and the dose of prednisone was reduced.

On 35th day after hospital discharge the follow-up Chest X-ray revealed rare interstitial changes with outstanding improvement in comparison to the previous examination (Fig. 1).

### The control at the neurological outpatient clinic

On 23th day after hospital discharge due to the problems with walking noticed by the patient and his family, he went to the Neurological Outpatient Clinic (Fig. 1). The patient complained of numbness of toes and involuntary muscle spasms in hands, which appeared after discharge from the Infectious Diseases Ward.

Neurological examination revealed asymmetry of pupils – Adie syndrome (R > L, the pupillary light reflex and the accomodation-convergence reflex slower in the affected eye, photophobia, more miotic response in the eye after dilute pilocarpine test), weakened muscle strength of the right upper limb, decrease of deep tendon reflexes in lower limbs, decrease of exteroceptive sensation in right hand and toes, bilateral absence of quadriceps reflex, plantar reflex and reduced ankle-jerk reflex, steppage gait and bilateral peroneal nerve damage. Patient was diagnosed with bilateral paralysis of peroneal nerve.

**Fig. 4:**
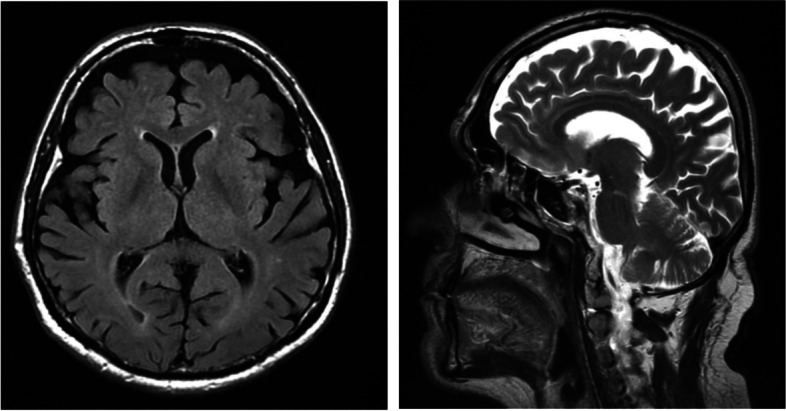
MRI of the head in the T1 sequence without contrast (**A**) revealed supratentorial and infratentorial cortical atrophy, retrograde vascular lesion (Fazakes scale: grade 1) and in the T2 sequence (**B**) without contrast revealed partial empty sella syndrome

A cranial and spine magnetic resonance imaging (MRI) on 36th day after hospital discharge revealed supratentorial and infratentorial cortical atrophy, partial empty sella syndrome, retrograde vascular lesion (Fazakes scale: grade 1), discopathy in the cervical and lumbosacral (L2-S1) segment with stenosis of a spinal canal (L2-S1) (Fig. 4). The patient has no previous history of imagining such as cranial and spine MRI. It is unknown if infratentorial cortical atrophy and partial empty sella syndrome had previously existed.

One month after diagnosis of peroneal nerve palsy (10.12.2021 r.) general condition of patient improved, but in physical examination he had still presented an unsteady, steppage gait. Neurophysiological examination revealed bilateral axonal mononeuropathy of the peroneal nerves accompanied by radiculopathic changes. The treatment consisted of motoric rehabilitation, orthotic footwear and control of the glucose level. Besides that, has continued steroid therapy (with scheme of doses reduction) and anticoagulant treatment (with rivaroxaban).

## Discussion and conclusions

Here we present the case report which has developed neurological complications after COVID-19. The most common symptoms associated with SARS-CoV 2 infection are fever, cough, headache, fatigue, myalgia, dyspnoea. They may evolve into pneumonia, respiratory insufficiency or even multiple organ failure [4]. Severe neurological complications are less frequent, but may occur because the virus has affinity for neural tissue [5]. To the most common early neurological manifestations belong the loss of smell and taste. One of European studies showed that 85,6% of infected patients experienced olfactory dysfunctions and 88% of them had gustatory disorders [6].

Our patient developed bilateral paralysis of peroneal nerve secondary to COVID-19. Peroneal nerve palsy is a common lower extremity neuropathy. The main causes include fracture of the fibula, direct trauma, knee torsion trauma or sitting in the kneeling position for many hours. To the others belong chronic neuropathies, i.e. degenerative changes in the spine, metabolic diseases (diabetes), or presence of atherosclerotic deposits that impair proper circulation. Compression is the most common cause. Our patient did not experience any of the above. We have excluded positional ischemia as a possible cause of neuropathy because the patient was not in the prone position for a long time. Impaired glucose tolerance also does not seem be the cause of neuropathy. Due to an episode of thrombosis it is possible that thrombosis of the microcirculation of the axon induced neuropathy. There are also hypotheses that SARS-CoV 2 attaches to ACE2, which are located on many human cells including neurons. Neuropathies could also be caused by cytokine storm, which is the response of immune system to viral infection [7].

The SARS-CoV 2 may invade both central and peripheral nervous system (CNS, PNS). Reports of COVID-19-related neuropathies, including symmetrical lower motor neuron quadriparesis, lower limb areflexic weakness, gait ataxia and quadriparesis, were published. Neurological symptoms in those cases had usually developed around 12–20 days after the onset of infectious symptoms [8]. Guillain-Barré syndrome secondary to COVID-19 is another disease, which seems to be the most commonly described PNS disorder. It is an acute polyradiculopathy with progressive, symmetrical limb weakness, areflexia, sensory symptoms and sometimes facial weakness. Some patients have developed the Miller Fisher variant of Guillain-Barré syndrome after SARS-CoV 2 infection. It is characterized by ophthalmoplegia, ataxia, and areflexia [9]. Described manifestations concerning PNS are also facial nerve palsy [10] and cranial nerve palsy resulting in opthalmoparesis [11].

There were described several CNS-related manifestations associated with COVID-19, including encephalitis, encephalopathies, acute disseminated encephalomyelitis (ADEM) and myelitis [9, 12, 13]. In some cases the CNS invasion was proved by CSF analysis – the specific RNA of the SARS-CoV-2 was detected [9, 13].

Some of COVID-19 patients have developed cerebrovascular manifestations such as ischaemic stroke, intracerebral haemorrhage or cerebral venous sinus thrombosis. Patients with severe SARS-CoV 2 infection often present elevated levels of D-dimer and severe platelet reduction. For this reason, they have a tendency for clot formation. That may result in venous thromboembolism [12, 14]. Our patient had presented the same abnormalities in laboratory findings and was diagnosed with thrombophlebitis.

Several cases of de novo epileptic seizures in patients with COVID-19 were described [15]. There are different hypotheses about its occurrence. It may be secondary to the virus invasion to the brain, activation of glutamate receptors in cytokine storm or may be caused as an adverse effect of antiviral drugs [13, 16, 17].

Apart from neurological complications, that appear during the acute period of COVID-19 infection, there are also manifestations that reveal much later – the so-called long COVID-19. It demonstrates as easy muscle fatigue, moderate breathlessness, cognitive fog, headaches or psychiatric disorders [18]. Headache might be relevant prognostic factor for COVID-19 [19]. Tan et al. noted the existence of post-infection fatigue and cognitive 'fog' after the infection. What is more, postviral fatigue is characteristic for other viral infections, including the coronaviriade such as SARS [20]. Although some correlations between COVID-19 and these neurological symptoms are still unclear, we have to remember that they often accompany patients for months after discharge from the hospital, decreasing the quality of their life.

Taking that all into consideration, it turns out, that neurological manifestations can precede any other symptoms of COVID-19 and sometimes may remain the only manifestation of the disease. Moreover, the occurrence of neurological complications is impossible to predict.

At the moment, we do not have the appropriate treatment for neurological complications of COVID-19, vaccines seem to be the only way to overcome them. All the more we need to insist on the social awareness and to reduce the reluctance of the public to the vaccination.

In conclusion, the present case stress the importance of neurological assessment of patients with COVID-19. We have presented a unique event of bilateral paralysis of peroneal nerve. There are many reports of SARS-CoV 2 infection associated with invasion to neural tissue. Patients may present diverse neurological symptoms. Although severe neurological complications of COVID-19 are not as common as respiratory disease, the number of patients that will develop neurological problems, will be increasing due to the continuing pandemic. Some of them will be forced to face the lifelong disability.

## Data Availability

All data related to this case report are documented within this manuscript.

## Abbreviations

COVID-19: Coronavirus disease 2019
SARS-CoV 2: Severe Acute Respiratory Syndrome Coronavirus 2
CRP: C-reactive protein
CXR: Chest X-Ray
CT: Computed Tomography
AspAT: Aspartate Transaminase
GGTP: Gamma-glutamyl Transferase
PCT: Procalcitonin
OGTT: Oral Glucose Tolerance Test
ECG: Electrocardiogram
LMWH: Low molecular weight heparin
RT-PCR: Reverse transcriptase-polymerase chain reaction in real time
USG: Ultrasonography
MERS: Middle East Respiratory Syndrome
CNS: Central nervous system
PNS: Peripheral nervous system
ADEM: Acute disseminated encephalomyelitis
